# Focus on degenerative retinal disorders

**DOI:** 10.1515/medgen-2024-2063

**Published:** 2025-02-12

**Authors:** Heidi Stöhr, Bernhard H. F. Weber

**Affiliations:** University of Regensburg Institute of Human Genetics Franz-Josef-Straus-Allee 11 93053 Regensburg Germany; University Regensburg Institute of Human Genetics Franz-Josef-Straus-Allee 11 93053 Regensburg Germany

More than two decades ago, a special issue of the *medizinische genetik* was devoted to retinal disorders, titled “Retinal Degeneration – From Clinic to Genetics to Therapy” (*medgen* 15, 2003). At that time, the diagnosis of inherited retinal dystrophies often presented a challenge, hampered by the difficulty of distinguishing between different retinal disease patterns. Advanced imaging and electrophysiology methods were largely in the experimental stage and often limited to specialized ophthalmic centers. Our knowledge of the factors responsible for single-gene retinopathies was based on about 80 identified disease-linked genes – a number that, while providing important initial insights, now seems modest compared to our current understanding. The genetics of more complex retinal diseases, such as glaucoma or age-related macular degeneration (AMD), were poorly understood until a breakthrough in 2005, when genetic variants in the complement factor H gene were identified as risk factors for AMD. During this time, treatment strategies focused on conventional methods, including visual aids, nutritional supplements, and complicated surgical techniques.

In the years that followed, remarkable progress has been made across the spectrum of retinal disease, improving clinical diagnosis, gene identification, DNA testing, relapse risk assessment, and personalized therapies. Optical coherence tomography (OCT) has transformed retinal imaging, providing non-invasive, high-resolution cross-sectional views of the retina. This, together with other specialized techniques, allowed precise in vivo examination of different retinal layers, from the innermost retinal nerve fibre layer to the outermost retinal pigment epithelium. The number of disease genes for retinopathies has steadily increased, allowing for detailed molecular genetic diagnostics with a high degree of accuracy, ultimately paving the way for gene- and mutation-specific treatments tailored to individual needs. Reflecting these advances, a follow-up special issue of the *medizinische genetik*, titled “Therapeutic Strategies for Hereditary Retinal Diseases” (*medgen* 2, 2017) touched on these topics, highlighting the role of the breakthroughs at the forefront of translational research and their potential impact on precision medicine across various clinical areas.

The latest special issue of the *medizinische genetik*, “Focus on Degenerative Retinal Disorders”, continues this exploration by addressing key elements critical to stratified patient care. It highlights important developments in genetic counselling, DNA diagnostics, methods of drug delivery to the retina, and treatments approved by regulatory bodies such as the FDA and EMA, highlighting the importance of advancing patient-specific therapeutic approaches.

The articles by *Stöhr and Weber* and *Kellner* et al. summarize the current state of DNA testing and highlight the diagnostic challenges posed by the known clinical and genetic heterogeneity of inherited retinal disorders. DNA diagnostics has progressed rapidly from single gene analysis to multigene panel testing, whole exome sequencing and, more recently, to whole genome sequencing. *Stöhr and Weber* particularly focus on the use of the latter advanced technology and critically discuss the results of studies published in recent years. *Kellner* et al. provide an ophthalmologist’s perspective on the clinical importance of DNA diagnostics and the direct benefits for patients in daily life and career decisions. Their article highlights the wide range of clinical presentations of retinal dystrophies and outlines approaches to clinical diagnosis and diagnostic confirmation.

*Cornelis and Cremers* re-evaluate an earlier model for estimating recurrence risk in autosomal recessive ABCA4-associated Stargardt disease, recognizing that disease-contributing alleles vary widely in their functional effects and phenotypic expression. They now incorporate additional factors such as structural variants, de novo mutations, penetrance, sex, and ethnicity into an updated model, improving the accuracy of relapse risk calculations. This refined approach not only benefits Stargardt disease counselling but may also help to improve risk assessment for other autosomal recessive disorders.

Extending the scope to complex non-Mendelian retinal diseases such as age-related macular degeneration (AMD), *Kiel and Weber* discuss the use of polygenic risk scores (PRS) derived from genome-wide association studies. They caution against using PRSs for predictive testing due to uncertainties in assessing genetic risk assessment but acknowledge their utility in confirming diagnoses when clinical symptoms are present, and treatment options are available.

The final three articles examine some of the therapeutic challenges in retinal degeneration. *Klaus et al.* address the difficulties and limitations of ocular drug therapy targeting the posterior pole of the eye, a region that is difficult to access due to the complex anatomy and physiology of the human eye. They critically evaluate the advantages and disadvantages of drug delivery using nanotherapeutic approaches. *Lorenz* reviews the long-term outcomes of gene transfer therapy for RPE65-associated retinal diseases, highlighting important concerns such as the risk of accelerated chorioretinal atrophy, which may diminish the perceived efficacy of this unique therapy. Finally*, Zeng et al.* discuss the approved use of idebenone, a potent intramitochondrial antioxidant, for the treatment of Leber hereditary optic neuropathy (LHON), a mitochondrial disorder resulting from complex I dysfunction in the respiratory chain. The authors also review intravitreal gene replacement therapies for LHON, several of which have completed late-stage clinical trials.

The editorials of the 2003 and 2017 special issues both emphasized the importance of “molecular medicine” in providing precision medicine for patients with inherited retinal diseases. Starting in 2024, Germany will launch a five-year pilot project on genomic medicine, aiming to improve medical care by integrating genomic data and clinical information. This development offers hope to patients with severe impairment caused by inherited retinal diseases – many may even see the proverbial light at the end of the tunnel in their lifetime.

